# Sublingual Immunization With an RSV G Glycoprotein Fragment Primes IL-17-Mediated Immunopathology Upon Respiratory Syncytial Virus Infection

**DOI:** 10.3389/fimmu.2019.00567

**Published:** 2019-03-28

**Authors:** In Su Cheon, Joo Young Kim, Youngjoo Choi, Byoung-Shik Shim, Jung-ah Choi, Dae-Im Jung, Jae-Ouk Kim, Thomas J. Braciale, Hyewon Youn, Man Ki Song, Jun Chang

**Affiliations:** ^1^Laboratory Science Division, International Vaccine Institute, Seoul, South Korea; ^2^Graduate School of Pharmaceutical Sciences, Ewha Womans University, Seoul, South Korea; ^3^The Beirne B. Carter Center for Immunology Research and Department of Pathology, The University of Virginia, Charlottesville, VA, United States; ^4^Department of Nuclear Medicine, Cancer Research Institute, Seoul National University College of Medicine, Seoul, South Korea; ^5^Cancer Imaging Center, Seoul National University Hospital, Seoul, South Korea

**Keywords:** RSV, glycoprotein, sublingual, IL-17, immunopathology

## Abstract

Respiratory syncytial virus (RSV) is the leading cause of serious respiratory tract disease but there is no licensed RSV vaccine. Immunopathological mechanisms have long been suspected as operating in the development of severe RSV disease and have hampered the development of safe and effective vaccines. Here, we show that unlike intranasal immunization, sublingual immunization with RSV glycoprotein fragment containing the central conserved region (Gcf) primes the host for severe disease upon RSV challenge. This increased pathology does not require replication by the challenge virus and is associated with massive infiltration of inflammatory cells, extensive cell death, and excessive mucus production in the airway and lungs. This exacerbated RSV disease primed by sublingual Gcf immunization is distinct from the immunopathology by G-expressing vaccinia virus or formalin-inactivated RSV, and preceded by prominent IL-17 production. IL-17 deficiency abolished the enhanced disease. Our results suggest a novel mechanism of RSV vaccine-induced immunopathology by IL-17, and highlights the importance of vaccination site.

## Introduction

Human respiratory syncytial virus (RSV) is a negative sense, single-stranded RNA virus belonging to the Paramyxovirus family. RSV infection is the leading cause of lower respiratory tract disease in infants and young children, and elderly worldwide (1, 2). However, there is no safe and effective vaccine presently licensed for human use. In the 1960s, children that received formalin inactivated RSV (FI-RSV) vaccine experienced severe enhanced disease, characterized by extensive pulmonary inflammation and eosinophilia, upon subsequent natural infection with RSV (3, 4). Similarly, mice immunized with a recombinant vaccinia virus expressing RSV G protein (vvG) also experienced extensive pulmonary inflammation and pulmonary eosinophilia upon RSV infection, mimicking the enhanced disease in children that received FI-RSV vaccination (5). The vaccine-enhanced disease caused by FI-RSV or vvG vaccination was also typified by extensive secretion of Th2 cytokines, and abrogating the functions of these cytokines reduced disease severity in both models (6, 7). The similar disease characteristics of pulmonary eosinophilia and Th2-biased cytokine response following RSV challenge in FI-RSV- and vvG-vaccinated subjects implicated RSV G protein as the likely cause of disease enhancement (8, 9). Accordingly, subsequent studies have revealed that RSV G protein possesses immuno-modulatory properties capable of altering the immune response in the RSV infected host (10–12). For example, it has been reported that RSV G protein inhibits the development of an innate immune response normally elicited by the virus and endotoxin (12). Johnson et al. also reported that priming with secreted form of G glycoprotein augmented IL-5 production and tissue eosinophilia after RSV challenge (10). Another mechanism of immune modification adopted by RSV G protein includes “chemokine mimicry” utilizing the CX3C chemokine-like motif (aa 182-186) within its central conserved region of G, which contains marked similarity to the receptor binding region of fractalkine, CX3CL1, thereby mimicking fracktalkine and interfering with leukocyte chemotactic activity (13, 14). Also, the immune response to a peptide corresponding to G_183−197_, (a known CD4^+^ T cell epitope within G protein) has been linked with severe pulmonary eosinophilia suggesting involvement of this T cell epitope in the disease enhancement (15).

In present study, we have identified a novel vaccination route dependent type of RSV vaccine-induced disease, caused by prior exposure to RSV G protein distinct in mechanism and features, from the enhanced disease produced by vaccination with FI-RSV or vvG. We report that sublingual administration of Gcf, a recombinant polypeptide corresponding to the central conserved fragment of the RSV G protein predisposes the immunized animals to enhanced pulmonary disease upon challenge with live RSV. The observed disease enhancement was characterized by prominent IL-17 production, massive infiltration of inflammatory cells, and excessive mucus production in the airway and lungs of the affected animals. Moreover, we show using Gcf vaccinated IL-17 knockout mice that IL-17 is prerequisite for this Gcf-mediated disease enhancement.

## Materials and Methods

### Materials

For cell culture and virus preparation, Dulbecco's Modified Eagle Medium (DMEM) and fetal bovine serum (FBS) were purchased from Life Technologies (Grand Island, NY, USA) and Lonza (Basel, Switzerland), respectively. To measure cytokine concentrations, cytometric bead array (CBA) mouse inflammation/Th1/Th2/Th17 cytokine kit, mouse IL-5 and IL-13 flex sets were purchased from BD Biosciences (San Diego, CA, USA). All reagents for flow cytometry including Golgi Plug, Cytofix/Cytoperm solution, anti-mouse CD16/32 (Mouse BD Fc Block^TM^), CD4-APC-Cy7, CD44-FITC, IFN-γ-APC, IL-17-PE, IL-5-APC, CD11c-FITC, CD45-PerCP, Siglec-F-PE, and Ly6G-PE-Cy7 were purchased from BD Biosciences. For development of ELISA, horseradish peroxidase (HRP)-conjugated goat anti-mouse IgG was purchased from Southern Biotechnology (Birmingham, AL, USA). HRP-conjugated goat anti-mouse IgG1 and IgG2a were purchased from Zymed Laboratories (San Francisco, CA, USA).

### Virus Preparation

RSV A2 strain was propagated in HEp-2 cells (ATCC, Manassas, VA, USA) grown in DMEM containing 3% of FBS. RSV replication was confirmed by formation of syncytia. Infected cells were harvested by scraping at day 3 or 4 post infection. Harvested cells were lysed by sonication, and virus particles were isolated via high-speed centrifugation. Virus titer was determined by standard RSV plaque assay.

### Preparation of Gcf

The plasmid containing genetic sequence for Gcf derived from RSV A2 G protein spanning from amino acid residues 131–230 was prepared as follows. Gcf plasmid was transformed into *Escherichia coli* BL21 (DE3) strain (Novagen, Madison, WI, USA). Transformed *E. coli* were grown overnight at 37°C in Luria-Bertani (LB) medium supplemented with 100 mg/ml of ampicillin for selection. Bacterial culture was transferred into fresh LB medium and cultured at 37°C while shaking at 180 rpm until OD_600_ of 0.6~0.8. Protein expression was induced by the addition of 0.5 M IPTG. *E. coli* were harvested by centrifugation at 6,000 rpm for 10 min. Bacterial pellets were suspended in binding buffer (20 mM Tris, 0.5 M NaCl, pH 7.9) and disrupted by sonication on ice. Soluble and insoluble fractions were separated by centrifugation for 40 min at 20,000 rpm. Soluble fractions were applied to a Talon metal affinity column, washed with binding buffer containing 20 mM imidazole, and then the proteins were eluted by using an elution buffer (300 mM imidazole, 20 mM Tris, 0.5 M NaCl, pH 7.4). The purified proteins were dialyzed in PBS. The endotoxin in each purified protein was removed by using Triton X-114 as previously described (16). The endotoxin level of each protein was measured by the limulus amebocyte lysate (LAL) assay kit according to the manufacturer's instructions (Lonza). Purified proteins were electrophoresed on 15% SDS-PAGE and the protein bands were visualized by staining with Coomassie Brilliant Blue (Bio-Rad Laboratories, Hercules, CA, USA). Protein concentration was determined by Bradford protein assay kit (Bio-Rad Laboratories).

### Mice and Immunization

Female BALB/c mice, 6–8 weeks old, were purchased from Orient Bio Inc. (Seoul, Korea). IL-17 knockout mice were provided by Yoon-Keun Kim (POSTECH, Korea). All mice were maintained under specific pathogen-free condition, and all studies were approved by Institutional Animal Care and Use Committee (IACUC) at International Vaccine Institute (Approval No. 2014-001). Mice were immunized with 20 μg of Gcf with 2 μg of CT (List Biological Lab. Inc. Campbell, CA, USA) via i.n. or s.l. route, and a booster immunization was administered 14 days after the primary immunization. For s.l. immunization, 20 μl of prepared antigen was gently placed underneath the tongue of the anesthetized mice. Following antigen delivery, mice were maintained with heads positioned in anteflexion for at least 30 min. For i.n. immunization, 50 μl of prepared antigen were administered into the left nostril of the anesthetized mice. Three weeks after the booster immunization, mice were challenged with 2–3 × 10^6^ PFU of live RSV A2. As control, mice were also immunized with 2 μg of CT sublingually, with 1 × 10^7^ PFU of vvG by scarification or with 1 × 10^6^ PFU of FI-RSV intramuscularly.

### Cytokines

Mice were immunized on days 0 and 14 and challenged with RSV A2 as described above. On expected days (0–9 days) post-challenge, mice were sacrificed, and Bronchoalveolar lavage (BAL) fluids and lungs were harvested. Cytokine levels in BAL fluid and lung homogenate were determined using CBA mouse inflammation/Th1/Th2/Th17 cytokine kit, mouse IL-5 flex set, and mouse IL-13 flex set according to the manufacturer's protocol.

### Lung Virus Titration

Lung virus titer was determined from the lungs harvested from mice at day 4 post challenge. Harvested lungs were passed through 70 μm cell strainer (BD Biosciences) in serum-free RPMI-1640. Lung supernatant was collected via centrifugation, and RSV titer in the lung supernatant was determined by plaque assay on HEp-2 cells.

### Detection of Eosinophils and Neutrophils in BAL and Lung

On expected days (0–9 days) post-challenge, mice were sacrificed and BAL fluids and lung samples were collected and cells were isolated from the BAL fluid or lung supernatant by centrifugation. Isolated cells were resuspended in FACS staining buffer (PBS with 1% FBS) and stained with CD11c-FITC, CD45-PerCP, Siglec-F-PE, and Ly6G-PE-Cy7 in the presence of anti-mouse CD16/32. Analysis of cell surface marker expression was performed using a BD LSRII flow cytometer (BD biosciences) and FlowJo software (Tree Star, San Carlos, CA, USA). A total of 100,000 events were analyzed per sample. Based on cell surface markers expression two different cell type were identified: CD45^+^, CD11c^−^, SiglecF^+^ cells as eosinophils and CD45^+^, CD11c^−^, Ly6G^+^ cells as neutrophils.

### Expression of T-bet, GATA-3 and RORγt in CD4 T Cells

Fourteen days after the booster immunization, immunized mice were sacrificed, spleens were harvested, and single cell suspension was prepared by passage of spleen samples through 70 μm cell strainer (BD Biosciences) in serum-free RPMI-1640. To examine the expression of t-bet (Th1), GATA-3 (Th2), and RORγt (Th17) transcription factors, cells were stimulated with 10 μg/ml of RSV G peptide corresponding to the amino acid (aa) sequence 183–195 (WAICKRIPNKKPG) for 18hr at 37°C. Cells were stained with anti-mouse CD4-APC-Cy7 and CD44-FITC. Cells were then fixed, permeabilized using Cytofix/Cytoperm solution, and further stained with anti-mouse T-bet-BV421, GATA-3-PE, and RORγt-APC and anti-mouse IL-17-PE or anti-mouse IL-5-APC. The cells were analyzed using BD LSRII flow cytometry and FlowJo software.

### ELISA for Detection of Antibodies

Levels of Gcf- or RSV A2-specific antibodies in the sera and BAL fluids were detected by ELISA. In brief, 96-well plates (Nunc, Roskilde, Denmark) were coated overnight with 100 μl of 2 × 10^4^ PFU/ml of purified RSV A2 diluted in 0.05 M carbonate-bicarbonate buffer at 4°C. After blocking with PBS containing 5% dried-skim milk for 1 h at room temperature, serially diluted serum or BAL fluid samples were added into the plate and incubated for 1 h at 37°C. Gcf- or RSV A2-specific antibodies were detected with HRP conjugated antibodies specific for mouse IgG, IgG1, IgG2a, or IgA followed by addition of TMB substrate for development. The absorbance at wavelength 450 nm was measured, and the endpoint titer was determined using O.D. cut-off values of 0.2.

### Histology

For histological analysis, mice were sacrificed at day 4 post-challenge and lungs were harvested following perfusion with 10 ml of heparinized PBS. Harvested lungs were fixed in 4% formalin for 48 h, embedded in paraffin, sectioned. Staining with hematoxylin and eosin (H&E) or periodic acid-Schiff (PAS) was performed to demonstrate inflammation and mucus production, respectively. Pathological score assigned representing the inflammatory cell infiltration shown in H&E staining and PAS positive cells per millimeter of bronchial basement membrane (mmBM) were measured by MetaMorp 4.6 (Universal imaging, Downingtown, PA, USA). TUNEL assay was performed using *In Situ* Cell Death Detection Kit (Roche, Mannheim, Germany) according to the manufacturer's protocol.

### Fluorescence Imaging

To assess the localization of antigens after their application, the anesthetized mice were injected with 5 μg of Alexa Fluor® 647-streptavidin conjugate (Invitrogen) via i.n. or s.l. route. The distribution of the fluorescence dye was monitored by *in vivo* imaging system (IVIS Lumina III; PerkinElmer Health Sciences, Waltham, MA) with Ex/Em of 640 nm/710 nm at 0.5, 1, and 24 h following injection. The average photon radiance on the certain surface of a mice was expressed as photons *per second* per centimeter squared per steradian (p/s/cm^2^/sr).

### Statistical Analysis

All data were plotted as a mean ± standard error, and statistical differences were determined using GraphPad Prism version 7 software (GraphPad Software, Inc., La Jolla, CA, USA). Data were analyzed for significance using an unpaired two-tailed Student's *t*-test, one-way analysis of variance (ANOVA) or two-way repeated-measures ANOVA with Tukey *post-hoc* test for group comparisons. The difference was considered statistically significant when the *P* < 0.05.

## Results

**Figure 1:**
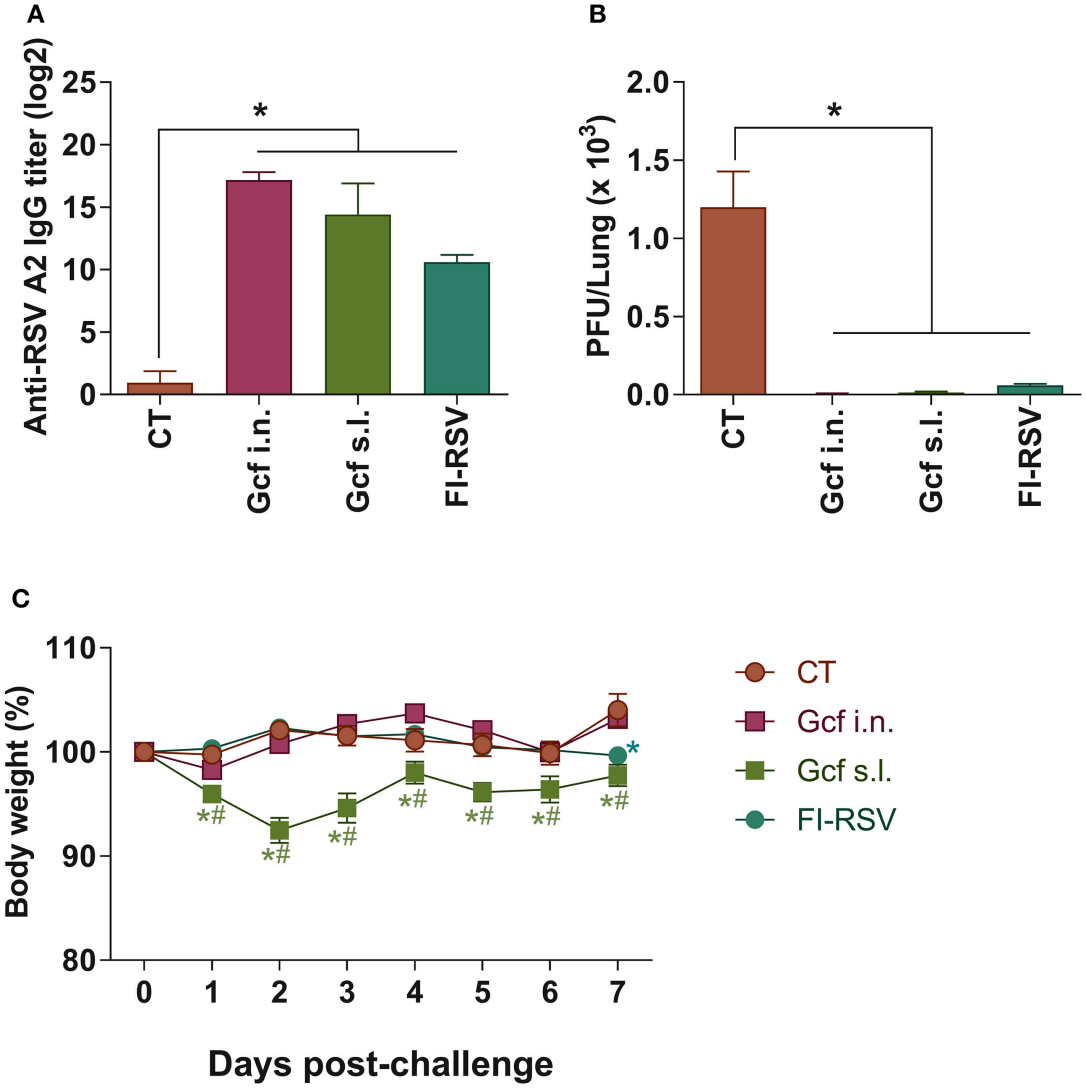
Anti-RSV immunity and enhanced disease in RSV challenged mice following sublingual Gcf immunization. **(A)** RSV-specific serum IgG titers measured at day 14 post-booster immunization by ELISA using 2 × 10^3^ RSV A2 per well as the coating antigen. Titers are indicated in log_2_. **(B)** Lung RSV titers at day 4 post-RSV A2 challenge determined by plaque assay. **(C)** Weight loss following RSV A2 challenge. Representative results of three independent experiments are shown. All data are expressed in mean ± S.E.M (*n* = 5). ^*^Significant different with CT group (*P* < 0.05). ^#^Significant difference with FI-RSV group (*P* < 0.05).

Recombinant polypeptide vaccine corresponding to the structurally conserved central core region of the RSV G protein (Gcf) has previously been developed (17). In an effort to evaluate the effectiveness of mucosal delivery of the polypeptide vaccine, we compared delivery by the intranasal (i.n.) or sublingual (s.l.) route of the Gcf polypeptide in a Cholera Toxin (CT) based adjuvant for protection against RSV infection. Sublingual immunization with CT alone was used as negative control, and FI-RSV- and vvG-immunization were used as controls to compare potential vaccine-enhanced disease which may arise from receiving an RSV G-based vaccine. There was a significant increase in the RSV-specific serum IgG titers detected in animals immunized with Gcf via i.n. and s.l. routes (Figure 1A and Figure S1A). Upon challenge with live RSV, significant reduction in RSV titer was observed in the lungs of Gcf-, FI-RSV- and vvG-immunized animals indicating that Gcf immunization via both i.n. and s.l. routes effectively reduced lung virus titer (Figure 1B and Figure S1B). Surprisingly, however, unlike the animals that received intranasal Gcf immunization, animals that received sublingual Gcf immunization suffered severe weight loss following RSV challenge (Figure 1C and Figure S1C). These observations suggested that prior exposure to this central conserved fragment of RSV G protein delivered via sublingual mucosa primes the host for enhanced morbidity upon subsequent RSV infection with a morbidity profile. Further, these data suggested that enhanced morbidity observed in Gcf s.l. group animals following RSV challenge was not caused by differences in antibody levels or the efficiency of virus clearance at the site of infection.

**Figure 2:**
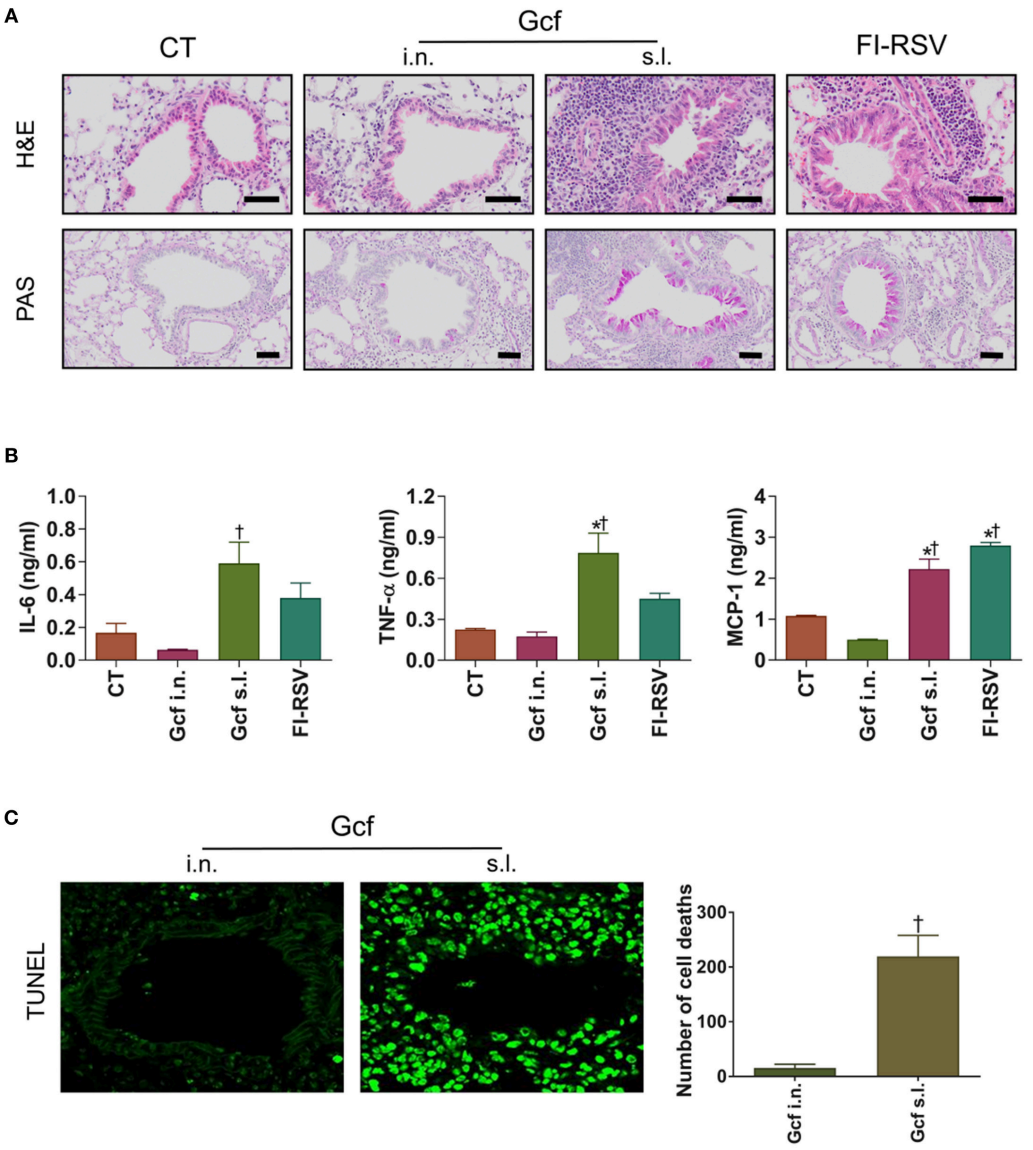
Lung histology and inflammatory cytokines of mice immunized with Gcf through different routes. All samples were collected at day 4 post-RSV challenge and prepared from formalin-fixed, paraffin-embedded lung tissues. H&E staining, TUNEL assay, and PAS staining were performed to determine inflammation, cell death, mucus secretion, respectively. The BAL fluid was collected at day 2 post-RSV challenge and performed CBA assay for the analysis of cytokines. **(A)** H&E staining (1st row, 20X magnification) and PAS staining (2^nd^ row, 20X magnification) of lung samples harvested at day 4 post-challenge. Scale bars indicate 50 micrometer. **(B)** IL-6, TNF-α, and MCP-1 levels in the BAL fluids. **(C)** TUNEL^+^ cells (40X magnification) and quantification of cell death represented by TUNEL^+^ cells. Representative results of three independent experiments are shown. All data are expressed in mean ± S.E.M (*n* = 3). ^*^Significant difference with CT group (*P* < 0.05). ^*†*^Significant difference with Gcf i.n. group (*P* < 0.05).

Next, to determine if enhanced morbidity in Gcf s.l. group, was reflected in the extent of lung injury, lung tissues were harvested at day 4 post RSV challenge and evaluated by light microscopy. Inflammatory cell infiltrations in the peribronchial and perialveolar regions were conspicuous in all RSV-challenged animals. However, the degree of inflammatory cell infiltration was markedly higher in the animals that received sublingual Gcf immunization, FI-RSV immunization and vvG immunization than in the animals that received intranasal Gcf or CT immunization (Figure 2A and Figures S2A,B). Inflammatory cytokines such as IL-6, TNF-α and MCP-1 were also higher in the animals that received sublingual Gcf immunization and FI-RSV immunization than in the animals that received CT immunization (Figure 2B). In addition, detection of cell death by TUNEL assay revealed extensive cell deaths in the airway and lungs of Gcf s.l group animals (Figure 2C). Increased cell death was also seen in RSV challenged vvG-immunized animals (Figure S2C). Such extensive cell death was not observed following intranasal Gcf or CT immunization (Figure 2C and Figure S2C). Furthermore, goblet cell hyperplasia and excessive airway mucus secretion which are features of the response to RSV infection in FI-RSV and vvG primed mice was also detected by PAS staining in the airway of animals in sublingual Gcf-immunized animals following RSV challenge (Figure 2A and Figure S2A). Goblet cell hyperplasia and airway mucus secretion were minimal in challenged animals that received intranasal Gcf- or CT-immunization. Histopathologic scoring of lungs also indicated significant lung pathology exclusively in sublingual Gcf- and vvG-immunized animals undergoing challenge RSV infection (Figure S2B). Together, these data demonstrated that the enhanced pathology induced with RSV infection after sublingual Gcf immunization is associated with massive infiltration of inflammatory cells and cytokines, extensive cell death, and excessive mucus production in the airway and lungs upon RSV infection. In addition, the finding that different manifestations of disease were displayed in FI-RSV-, vvG- and sublingual Gcf-primed groups led us to surmise that FI-RSV, vvG and sublingual Gcf immunization may cause disease enhancement by distinctly different mechanisms.

**Figure 3:**
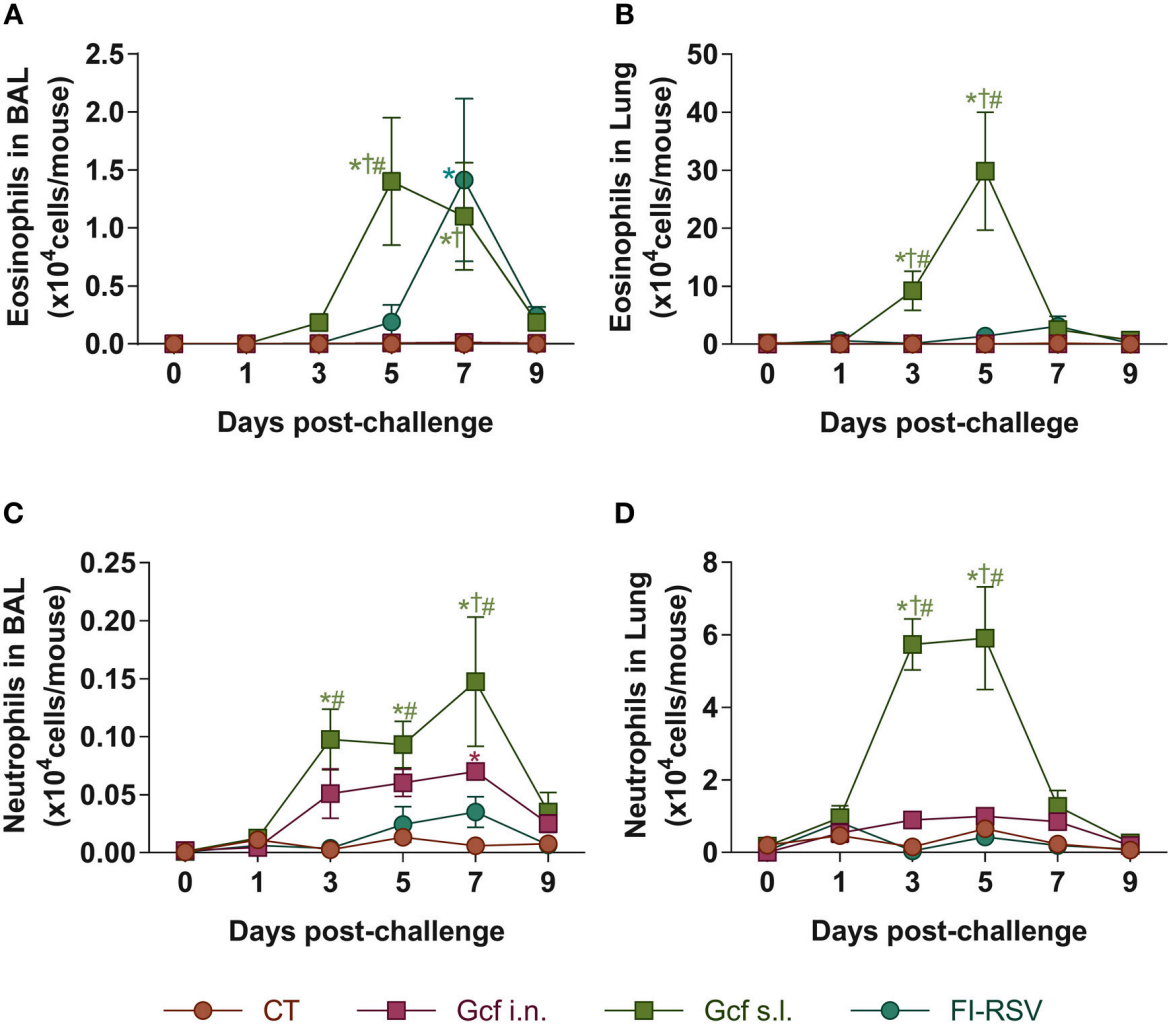
Eosinophil and neutrophil recruitment to the airways and lungs of Gcf-immunized mice following live RSV challenge. Eosinophil (CD45^+^CD11c^−^SiglecF^+^) count in **(A)** BAL fluids and **(B)** lung tissues after the RSV challenge. Neutrophil (CD45^+^CD11c^−^Ly6G^+^) count in **(C)** BAL fluids and **(D)** lung tissues after the RSV challenge. Representative results of three independent experiments are shown. All data are expressed in mean ± S.E.M (*n* = 6). ^*^Significant difference with CT group (*P* < 0.05). ^*†*^Significant difference with Gcf i.n. group (*P* < 0.05). ^#^Significant difference with FI-RSV group (*P* < 0.05).

Pulmonary eosinophilia is a well-known disease marker for RSV vaccine-enhanced disease (18, 19). Therefore, we examined eosinophil recruitment to the airway and lungs of Gcf- or FI-RSV-immunized animals following RSV challenge. As expected, we detected significant increase in BAL fluid eosinophil counts in FI-RSV-immunized animals (Figure 3A). Unexpectedly, BAL fluid eosinophil counts were also elevated in RSV challenged animals primed by sublingual Gcf immunization and at a level seven-fold higher than the increase detected in FI-RSV-immunized animals. Group-to-group statistical comparison confirmed significance of the difference in eosinophil count in Gcf s.l. group over the Gcf i.n. group (Figure 3A). These differences in eosinophil count between sublingual Gcf- and FI-RSV-primed mice undergoing challenge RSV infection were also evident in the infected lung parenchyma (Figure 3B).

At the same time, we also examined neutrophil recruitment to the airway and lungs. We detected significant increase in BAL fluid neutrophil counts in animals given sublingual Gcf immunization (Figure 3C). BAL fluid neutrophil counts were also elevated but to a lesser extent in challenged mice primed by Gcf via the i.n. route. Again, neutrophil count in the lungs among the immunization groups directly paralleled findings in the BAL fluid i.e., significantly increased neutrophil counts in the lungs of sublingual Gcf-primed animals over intranasal Gcf-primed animals (Figure 3D). Of note, we did not detect significant increase in neutrophil recruitment to the lungs in animals that received FI-RSV immunization compared to control CT primed and challenged mice. These results suggest that just as pulmonary eosinophilia characterizes vaccine-enhanced disease generated by prior immunization with FI-RSV, pulmonary neutrophilia appeared to be a prominent feature of enhanced disease produced by prior sensitization to RSV G protein introduced into the sublingual mucosa, and that the enhanced disease produced via sublingual immunization with G protein is distinctly different from the manifestation of disease exacerbation caused by FI-RSV immunization i.e., enhanced disease associated with the recall response to RSV induced by priming with FI-RSV characterized by pulmonary eosinophilia while priming to Gcf via the s.l. route results following RSV challenge in enhanced disease characterized by both pulmonary eosinophilia and neutrophilia.

**Figure 4:**
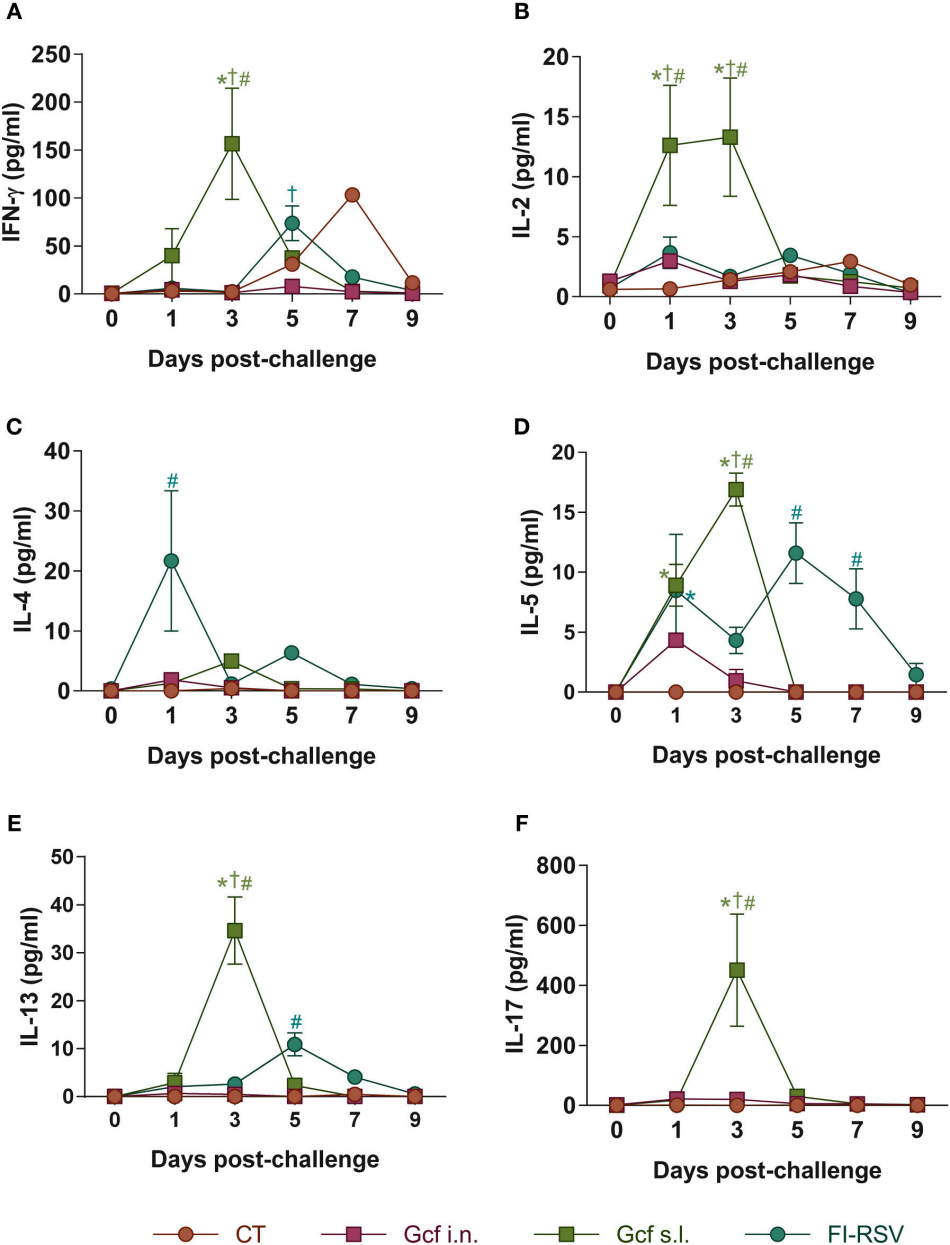
Cytokine profiles in the airway of mice immunized with Gcf. **(A–F)** IFN-γ, IL-2, IL-4, IL-5, IL-13, and IL-17 levels in BAL fluids of mice immunized with Gcf after RSV A2 challenge determined by CBA. Representative results of three independent experiments are shown. All data are expressed in mean ± S.E.M (*n* = 6). ^*^Significant difference with CT group (*P* < 0.05). ^*†*^Significant difference with Gcf i.n. group (*P* < 0.05). ^#^Significant difference with FI-RSV group (*P* < 0.05).

**Figure 5:**
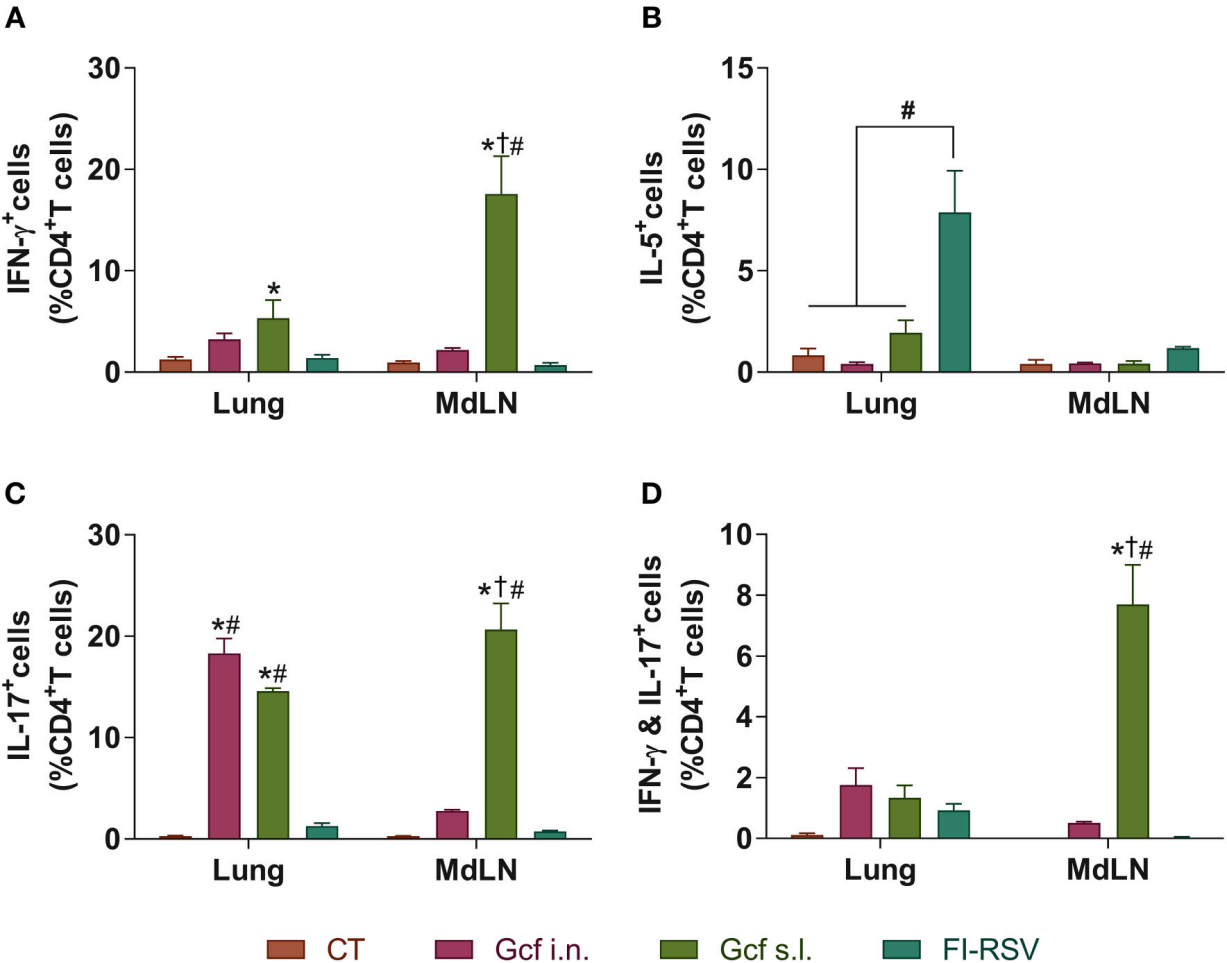
CD4 T cell responses in the mice immunized with Gcf. Percentage of **(A)** IFN-γ-, **(B)** IL-5-, **(C)** IL-17-, and **(D)** both IFN-γ and IL-17-producing CD4 T cells in the lung and mediastinal lymph node after RSV challenge determined by intracellular staining. Representative results of two independent experiments are shown. All data are expressed in mean ± S.E.M (*n* = 3). ^*^Significant difference with CT group (*P* < 0.05). ^*†*^Significant difference with Gcf i.n. group (*P* < 0.05). ^#^Significant difference with FI-RSV group (*P* < 0.05). *MdLD*, Mediastinal lymph node.

Next, to further examine the mechanism of enhanced disease caused by sublingual Gcf immunization, we examined cytokine levels in the airway and lungs of all immunized animals at 0~9 days post-challenge. BAL fluid IFN-γ, IL-4, IL-5, and IL-13 concentrations were significantly elevated in FI-RSV-immunized animals compared to animal given CT- or Gcf-immunization via either the i.n. or s.l. route. BAL fluid IFN-γ, IL-2, IL-5, and IL-13 concentrations were also significantly increased in Gcf s.l. group animals (Figure 4). With the significant increase in IL-17 concentration detected in the BAL fluids of animals in Gcf s.l. group (Figure 4F), the IL-17- and/or IFN-γ- producing CD4 T cells were also increased in the lungs and mediastinal lymph node of Gcf s.l. group following RSV challenge (Figure 5). Both Th1 and Th2 cytokines represented by IFN-γ and IL-4/5/13, respectively, were notably elevated in the BAL fluid of vvG-immunized mice, as previously described [Figure S3A; (20)], and IL-5 producing CD4 T cells were increased in the lung of FI-RSV-immunized mice (Figure 5B). However, we observed a significant increase in IL-17 levels in the BAL fluids and IL-17-producing CD4 T cells in the lungs and the draining lymph node of Gcf s.l. group animals, demonstrating the difference in the mechanism of disease exacerbation induced by FI-RSV, vvG and sublingual Gcf immunization. An exaggerated Th17 T cell-dependent cytokine response is most likely responsible for the enhanced RSV disease in animals given sublingual Gcf immunization, whereas, as our results and published findings (21) suggest, excessive Th1 and Th2 cytokine responses contribute to the enhanced RSV disease in animals primed by FI-RSV or vvG immunization.

We next analyzed the expression of canonical Th1, Th2, and Th17 transcription factors by intracellular staining in CD4 T cells in the spleens of immunized mice prior to RSV challenge. In keeping with the above findings, this analysis revealed significant increase in the percentage of RORγt expressing CD4 T cells in animals immunized with Gcf, while the percentages of t-bet (Th1) and GATA-3 (Th2) expressing CD4 T cells were increased in the spleens of vvG-immunized animals (Figure S3B). These results indicate that exposure to RSV G protein via the sublingual mucosa primes the host for Th17-biased immune response.

**Figure 6:**
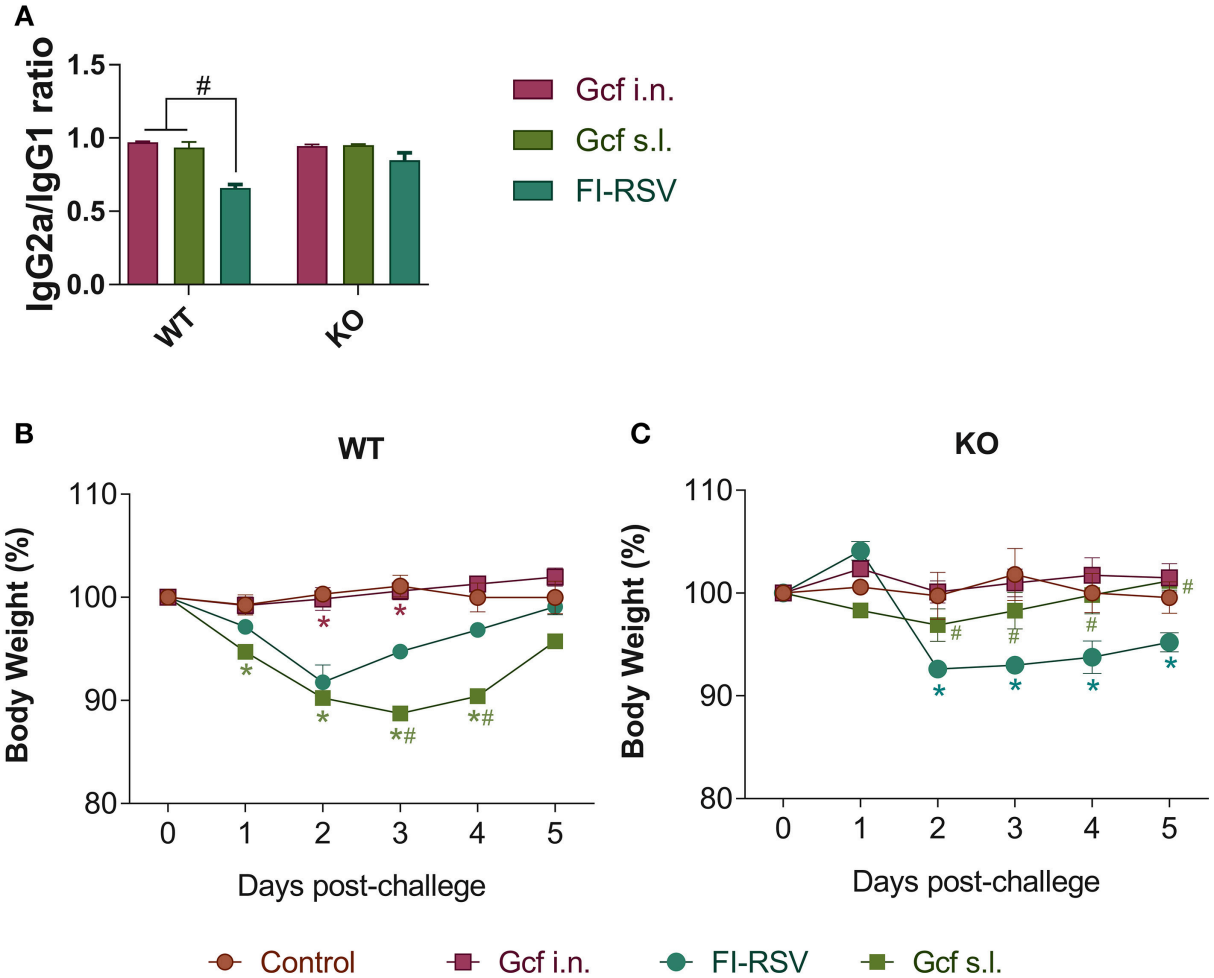
Effect of IL-17 on vaccine-induced immune response and weight loss following RSV challenge in mice immunized with Gcf. **(A)** Ratio of IgG2a/IgG1 in wild type mice and IL-17 KO mice immunized twice with Gcf via sublingual or intranasal route or with FI-RSV via intramuscular route. Sera were collected at 3 weeks after boost immunization. Both G-specific IgG2a and IgG1 were measured by ELISA and the ratio of IgG2a to IgG1 was calculated. ^#^Significant difference with FI-RSV group (*P* < 0.05). Body weight was monitored daily after RSV challenge in **(B)** wild type mice and **(C)** IL-17 KO mice immunized with Gcf or FI-RSV. Representative results of two independent experiments are shown. All data are expressed in mean ± S.E.M (*n* = 4). ^*^Significant difference with Control group (*P* < 0.05). ^#^Significant difference with FI-RSV group (*P* < 0.05). *WT*, wild type Balb/c mice; *KO*, IL-17 knock-out mice.

Next, we assessed whether a deficiency in IL-17 would affect the development of enhanced disease during RSV infection by using IL-17 deficient (KO) animals primed with Gcf via s.l. route. First, we determined Gcf-specific serum IgG2a/IgG1 ratio in order to determine Th1-Th2 bias. In WT animals, the IgG2a/IgG1 ratio was similar between the Gcf i.n. and s.l. groups (Figure 6A). However, the difference between serum IgG2a and IgG1 levels was significantly reduced in the FI-RSV group, indicating skewing of the immune response toward Th2. In IL-17 KO animals, the IgG2a/IgG1 ratios did not vary significantly between the immunization groups and there was no significant decrease in the IgG2a/IgG1 ratio in FI-RSV group in contrast to that observed in WT animals (Figure 6A).

**Figure 7:**
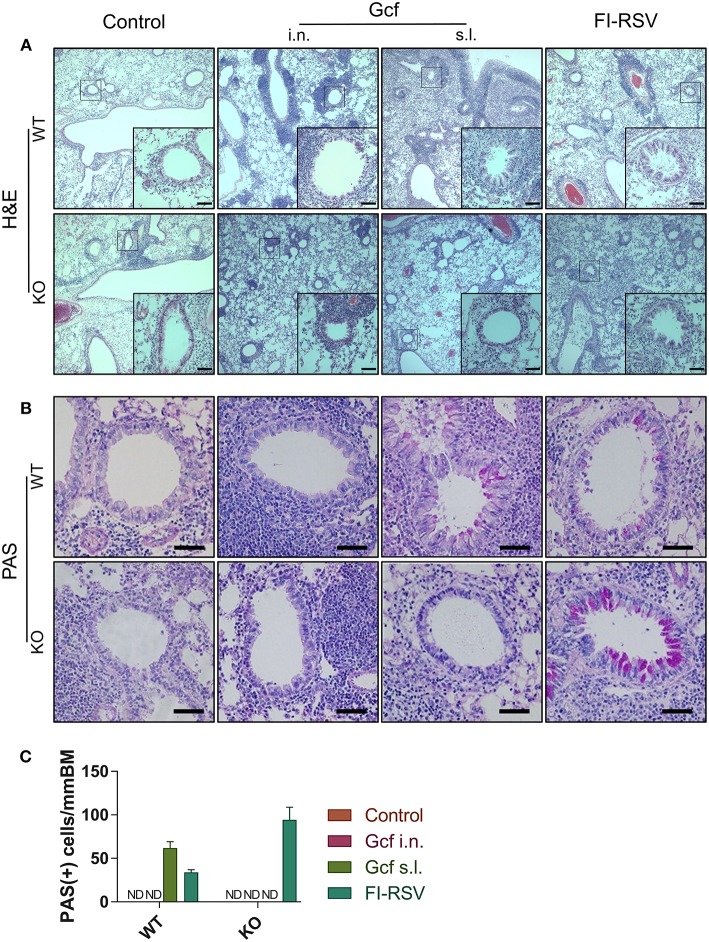
Effect of IL-17 on vaccine-induced histopathologic alterations in lung structure following RSV challenge. Mice were immunized twice with Gcf via sublingual or intranasal route or with FI-RSV via intramuscular route, and then challenged with RSV A2 strain. Histologic samples were prepared from formalin-fixed, paraffin-embedded lung tissues. **(A)** H&E staining and **(B)** PAS staining were performed and examined. **(C)** PAS-positive cells in peri-bronchial regions were measured for quantitative analysis. Representative results of two independent experiments are shown. All data are expressed in mean ± S.E.M (*n* = 4). Original magnification for **(A)** is 10X and the inset shows 40X. Original magnification for **(B)** is 40X. Scale bars indicate 50 micrometer in 40X. *WT*, wild type Balb/c mice; *KO*, IL-17 knock-out mice; *ND*, Not detected.

**Figure 8:**
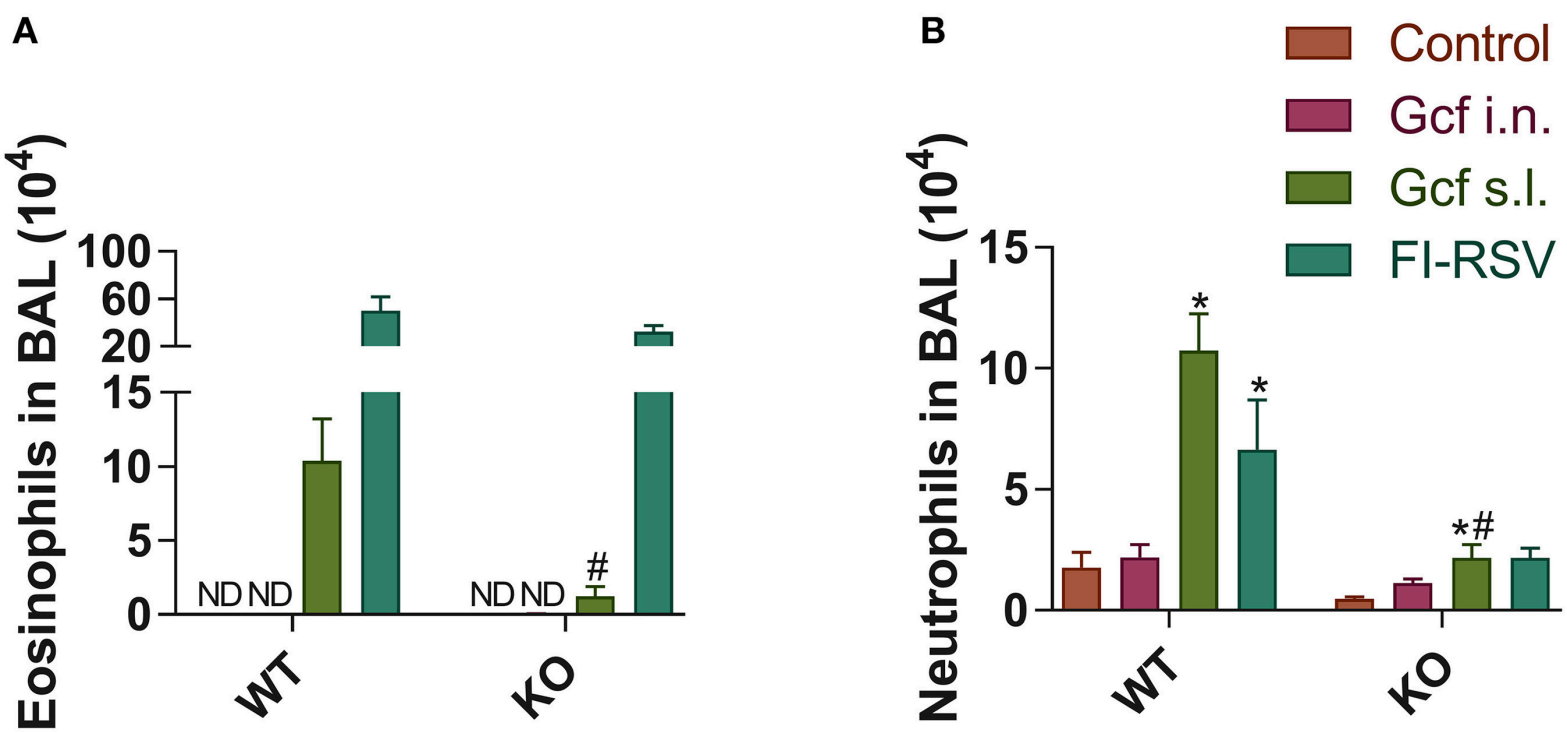
Granulocyte phenotypes in BAL fluid from wild-type and IL-17 KO mice immunized with different routes of Gcf after RSV challenge. BAL fluid cells were collected, counted and then analyzed by flow cytometry at 5 days after RSV challenge. **(A)** Eosinophils and **(B)** neutrophils in BAL fluid from wild type and IL-17 KO mice were measured in accordance with each of the criteria (CD45^+^ CD11c^−^ SiglecF^+^ and CD45^+^ CD11c^−^ Ly6G^+^, respectively). Representative results of two independent experiments are shown. All data are expressed in mean ± S.E.M (*n* = 4). ^*^Statistical significance with “Control” (*p* < 0.05). ^#^Statistical significance with “WT” (*p* < 0.05). *WT*, wild type Balb/c mice; *KO*, IL-17 knock-out mice; *ND*, Not detected.

We also observed a difference following RSV challenge in morbidity between WT and IL-17 KO mice as a function of prior immunization strategy. Among WT mice, animals immunized with Gcf s.l. or FI-RSV experienced more severe weight loss than animal that received no immunization or intranasal Gcf immunization (Figure 6B). The WT Gcf s.l. primed animals experienced prolonged weight loss and delayed weight recovery following RSV challenge, which was more pronounced even than in animals primed with FI-RSV. By contrast, among IL-17 KO mice, the Gcf s.l. primed animals experienced weight loss that was comparable to control unimmunized mice undergoing RSV infection (Figure 6C), suggesting a link between IL-17 expression and enhanced morbidity following RSV challenge in mice primed with Gcf via sublingual route. Histological analysis of infected lungs further supported a critical role for IL-17 in enhanced morbidity as there was a significant reduction, compared to WT IL-17 sufficient mice, in inflammatory cell infiltration and mucus secretion in the airway of IL-17 deficient Gcf s.l. primed animals following RSV challenge. Interestingly, severe inflammatory cell infiltration and excessive mucus secretion was still observed in FI-RSV immunized animals, even in the absence of IL-17 (Figure 7). Pulmonary eosinophilia was detected in FI-RSV group animals in both WT and IL-17 KO models (Figure 8). However, eosinophils as well as neutrophils were significantly reduced in the BAL fluids of IL-17 KO Gcf s.l. primed animals compared to the corresponding primed and challenged WT mice. In sum, these data strongly suggest a critical role of IL-17 as a mediator of enhanced disease following RSV infection in animals sensitized to RSV G via sublingual mucosa.

**Figure 9:**
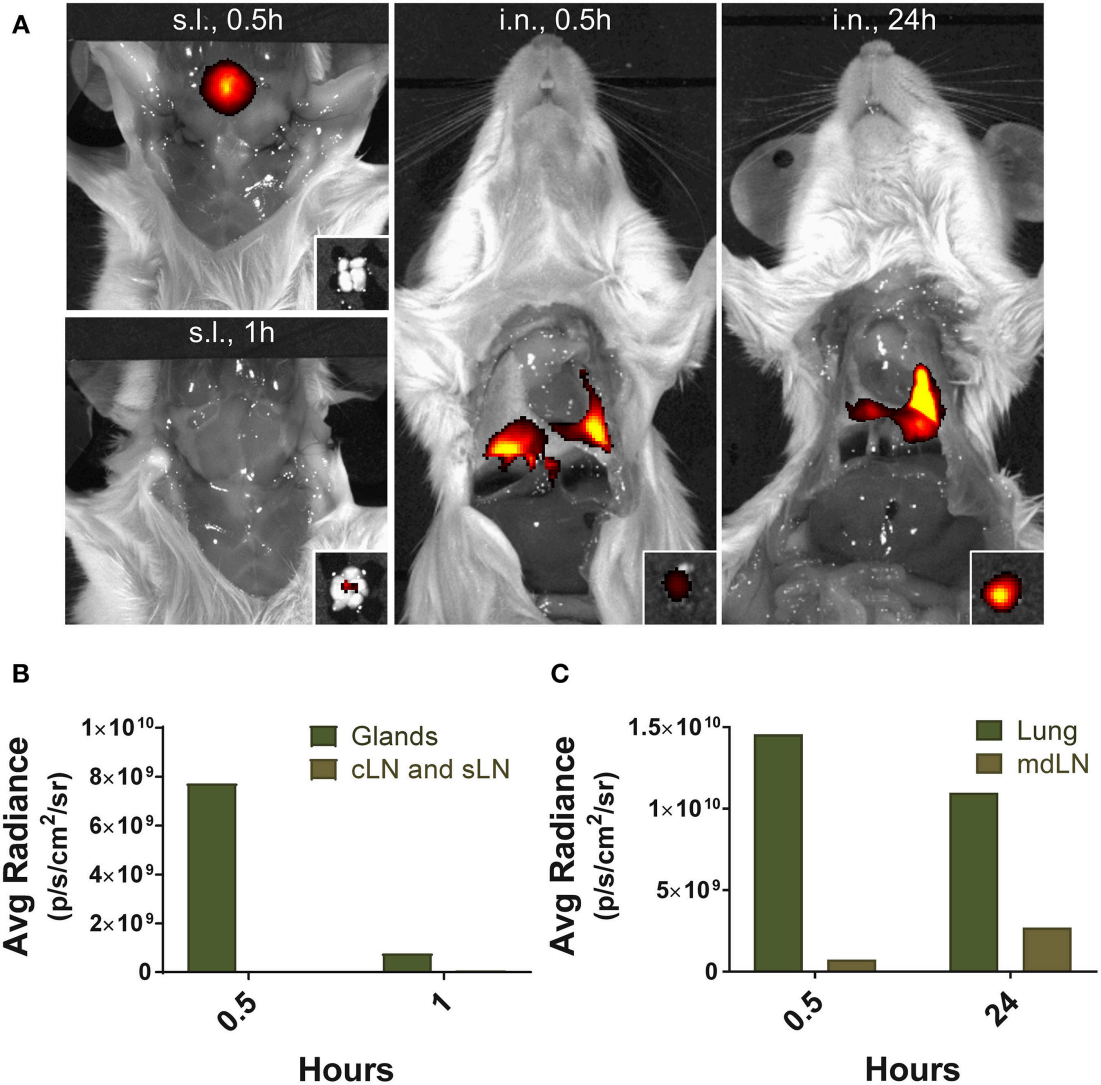
Localization of antigens following sublingual administration. Mice were anesthetized and injected with 5 μg of Alexa Fluor 647-streptavidin conjugate via s.l. or i.n. route. **(A)** Fluorescence images were measured by IVIS Lumina III with Ex/Em of 640 nm/710 nm at 0.5, 1, and 24 h following injection. *Small boxes*, draining lymph nodes from sublingual compartment or lung tissue. Average photon radiance of mice treated with the fluorescence dye via **(B)** s.l. and **(C)** i.n. route. Representative results of three independent experiments are shown. *Avg Radiance*, the average photon radiance on the certain surface of a mice was expressed as photons *per second* per centimeter squared per steradian (p/s/cm^2^/sr); *Gland*, Salivary gland; *cLN*, cervical lymph node; *sLN*, submandibular lymph node; *mdLN*, mediastinal lymph node.

Finally, to gain insight into the mechanisms leading to severe immunopathology by sublingual administration, we analyzed and compared the antigen trafficking following sublingual or intranasal administration using fluorochrome-conjugated protein. Fluorescence was detected near the tongue and salivary glands of the mice at 0.5 h after sublingual administration of Alexa Fluor 647 conjugated with streptavidin and fluorochrome-conjugated protein was no longer detectable within an hour (Figure 9). Draining lymph nodes around the sublingual mucosa, such as submandibular and deep cervical lymph nodes, showed no significant fluorescence during the indicated time period (Figures 9A,B). In contrast, in the case of intranasal administration, a large amount of fluorescence was observed in the lung tissue and the mediastinal lymph node at the same time point and even after 24 h (Figures 9A,C), suggesting that antigen trafficking and lymphatic drainage is different between sublingual and intranasal routes of immunization.

## Discussion

During our evaluation of various mucosal immunization routes to administer our RSV G-based subunit vaccine candidates, we unexpectedly observed that administration of Gcf via sublingual mucosa exacerbated disease severity upon live RSV challenge. Intranasal administration of the same antigen did not cause disease exacerbation, instead intranasal Gcf delivery conferred protection against RSV infection characterized by significant reduction in morbidity, lung virus titer, lung inflammation, and airway mucus secretion following live RSV challenge. Hence, because of the exacerbated disease severity we further investigated this immunization strategy as it related to the vvG and FI-RSV immunization models, which also have been shown to induce disease exacerbation upon RSV infection (22). First, our study demonstrated no correlation among the severity of disease, viral replication in the lung tissues, and the levels of RSV-specific antibodies distributed either systemically in the serum. The mice in immunization groups that produced similar RSV-specific serum IgG did not experience similar disease exacerbation. Further, our study demonstrated that quantity of live RSV in the lungs was not associated with the disease enhancement given that animals in CT group, even with their significantly higher lung virus titers, did not experience enhanced disease following challenge.

As expected, RSV challenge following FI-RSV or vvG-immunization induced pulmonary eosinophila; enhanced Th2-type responses such as increased airway and lung secretion levels of IL-4, IL-5, and IL-13; eosinophilic airway inflammation; and development of goblet cell hyperplasia and mucus hyperproduction. Elevation in airway secretion of IFN-γ was also simultaneously detected. Interestingly, in a previous study elevated production of IFN-γ was implicated in the increased clinical illness and airway resistance following RSV challenge of animals previously immunized with vvG (18). By contrast while a similar increase in eosinophil recruitment following RSV challenge in animals primed with sublingual Gcf there was also a, significant elevation in neutrophil recruitment and IL-17 secretion in the airway and lungs exclusively in the animals exposed Gcf via sublingual mucosa. Interestingly, disease characteristics in mice undergoing challenge following sublingual Gcf exposure with elevated neutrophil infiltration resemble the disease spectrum in subgroup of severe asthma described as “refractory” asthma. Of note in this regard a previous study demonstrated excessive neutrophilic infiltration to the airway of RORγt-overexpressing mice, with enhanced lung expression of IL-17A following exposure to the sensitizing antigen (23). We observed a remarkably similar disease pattern and cytokine profile in animals that received priming with Gcf by the sublingual route followed by RSV challenge.

It is also interesting to note that there are significant increases in both eosinophil and neutrophil recruitment to the BAL fluids and lung tissues of Gcf s.l. group post RSV challenge. This simultaneous elevation in eosinophil/neutrophil recruitment was unique to animals exposed Gcf via sublingual mucosa. This pattern of mixed granulocyte infiltration into the lungs following RSV challenge, suggests that s.l. priming with Gcf promotes an environment favoring IL-17 production. Consistent with this notion IL-17 has been shown to induce the release of eotaxin from airway smooth muscle cells as well as neutrophil influx (24). However, based on previous findings (18), it is unlikely that eosinophils contribute to the exacerbation of disease, although we observed significant increase in airway and lung eosinophil recruitment in Gcf s.l. group animals experiencing exacerbated disease.

The detrimental role of IL-17 in the development of inflammatory lung diseases such as asthma, chronic obstructive pulmonary disease, and cystic fibrosis has been well-documented (25). These evidences strongly suggest that IL-17 might play a prominent role in the pathogenesis of lung inflammation by promoting the recruitment of neutrophils. In our study, severe exacerbation of RSV disease characterized by induction of IL-17-biased immunity and pulmonary neutrophila/eosinophilia in mice that were previously exposed to RSV G protein via sublingual mucosa was observed. The presence of IL-17 in this Gcf-mediated disease exacerbation is critical since the absence of IL-17, reflected by IL-17 KO model, prevented the phenomenon. These suggest that IL-17 could play a prominent role in the pathogenesis of RSV G-based vaccine-enhanced disease.

The major source of IL-17 produced during the course of RSV infection was most likely Th17 cells because IL-17-producing CD4 T cells were increased in the lungs and mediastinal lymph node of Gcf s.l. group following RSV challenge (Figure 5) and the RORγt expression level was increased in splenic CD4 T cells following sublingual Gcf immunization (Figure S3). The resident memory CD4 T cells in the lung might produce IL-17 at early times during RSV infection (26). However, we cannot exclude the possibility that other immune cells such as γδ T cells and innate lymphoid cells (ILCs) could produce IL-17 upon RSV challenge (27). Another possibility is that novel IL-17-producing Th2-like cells might contribute to the Gcf s.l. immunization-induced vaccine enhanced diseases. Previously, it has been reported that antigen-specific inflammatory IL-17-producing Th2 cells promote influx of heterogeneous leukocytes including eosinophils and neutrophils and exacerbate allergic asthma (28).

Presently, sublingual mucosa is being targeted for sublingual immunotherapy (SLIT) for treatment of type 1 allergic hypersensitivity (13). Sublingual mucosa is also being extensively evaluated as a delivery route for various vaccines (29). These strategies to target sublingual mucosa for immunotherapy for allergies or as a vaccine delivery route have been broadly gaining momentum. Sublingual antigens might trigger the tolerogenic or immunogenic response depending on the type of antigen and adjuvant through local or systemic pathways (30, 31). Nagai et al. reported that sublingually administered antigens could be transported across epithelial cells in the sublingual ductal system to the ductal antigen-presenting cells within an hour. They also suggested that the sublingual duct, composed of pseudostratified and simple columnar epithelium could spread antigen rapidly through the paracellular and transcellular pathways, although mucosa-associated lymphoid tissues or M cell-like structures were not identified (32). In this study, we observed a rapid disappearance of sublingual antigen within 1 h near the tongue and salivary glands. In particular, it was rarely observed in the draining lymph nodes near the sublingual compartment, which is contrasted with the fact that nasal antigen remained in the mediastinal lymph node for up to 24 h after administration. These results suggest that the initial response to the sublingual antigen may be different from nasal administration.

Our findings represent a cautionary note regarding the use of sublingual route for antigen administration as SLIT or vaccination strategies. Although it is likely that the phenomenon observed in our study is specific to RSV G protein, we cannot exclude a possibility that other antigens, when administered via sublingual mucosa, could prime the recipient for an exacerbated disease upon subsequent re-exposure. In this regard, it is worth noting that increase in IL-17 mRNA expression in peripheral blood mononuclear cells (PBMC) was detected in children given SLIT for allergic rhinitis (33). Lastly, exposure to RSV G protein via different delivery routes appears to prime for T helper cell immunity that is differentially biased. For example, intramuscular FI-RSV immunization establishes Th2 bias (34), while vvG immunization via scarification primes for excessive Th1 and Th2 bias. Our findings reveal that sublingual delivery of RSV G institute Th17 bias. It is possible that such capability of inducing selective skewing of T helper immunity may potentially be used beneficially.

In conclusion, we report here a novel class of RSV vaccine-enhanced immunopathology which is primed by sublingual immunization and is regulated by IL-17. Understanding the mechanism of vaccine-enhanced disease in RSV infection is essential for the development of safe and effective vaccines, and our results demonstrating IL-17-mediated disease exacerbation provides a new model for evaluating safety of RSV vaccine candidates.

## Author Contributions

MS and JC designed and conceived the study. IC, JYK, YC, BS, J-aC, DJ, J-OK and HY performed the experiments. IC, JYK, YC, TB, MS, and JC wrote the manuscript. All authors reviewed the manuscript before submission, etc.

### Conflict of Interest Statement

The authors declare that the research was conducted in the absence of any commercial or financial relationships that could be construed as a potential conflict of interest.

## Supplementary Material

Figure S1 Anti-RSV immunity and enhanced disease in RSV challenged mice following sublingual Gcf immunization**. (A)** RSV-specific serum IgG titers measured at day 14 post-booster immunization by ELISA using 2 x 10^3^ RSV A2 per well as the coating antigen. Titers are indicated in log_2_. **(B)** Lung RSV titers at day 4 post-RSV A2 challenge determined by plaque assay. **(C)** Weight loss following RSV A2 challenge. Representative results of three independent experiments are shown. All data are expressed in mean ± S.E.M (*n* = 5). ^*^ indicates significant different with CT group (*P* < 0.05). ^#^ indicates significant difference with vvG group (*P* < 0.05). *ND*, Not detected.

Figure S2 Lung histology of mice immunized with Gcf through different routes. All samples were collected at day 4 post-RSV challenge and prepared from formalin-fixed, paraffin-embedded lung tissues. H&E staining, TUNEL assay, and PAS staining were performed to determine inflammation, cell death, mucus secretion, respectively. **(A)** H&E staining (1^st^ low, 20X magnification), and PAS staining (2^nd^ low, 40X magnification) of lung samples harvested at day 4 post-challenge. **(B)** Lung pathological score assigned representing the inflammatory cell infiltration shown in H&E staining. **(C)** TUNEL^+^ cells (40X magnification) and quantification of cell death represented by TUNEL^+^ cells. Representative results of three independent experiments are shown. All data are expressed in mean ± S.E.M (*n* = 3). ^*^ indicates significant difference with CT group (*P* < 0.05).

Figure S3 Cytokine profiles and transcription factor expression levels in the mice immunized with Gcf. **(A)** IFN-γ, IL-2, IL-4, IL-5, IL-13, and IL-17 levels in BAL fluid of mice immunized with Gcf at day 4 post-RSV A2 challenge determined by CBA. **(B)** Percentage of T-bet, GATA-3, or RORγt expressing splenic CD4+ T cells at day 7 post-booster immunization determined by intracellular staining. Representative results of three independent experiments are shown. All data are expressed in mean +/- S.E.M (*n* = 6). ^*^ indicates significant difference with CT group (*P* < 0.05). † indicates significant difference with Gcf i.n. group (*P* < 0.05). ND, Not detected.
