# Supplementary figures and images for: Sublingual Immunization With an RSV G Glycoprotein Fragment Primes IL-17-Mediated Immunopathology Upon Respiratory Syncytial Virus Infection
Source: Front Immunol. 2019 Mar 28;10:567. doi: 10.3389/fimmu.2019.00567 (PMC6447673; doi:10.3389/fimmu.2019.00567)

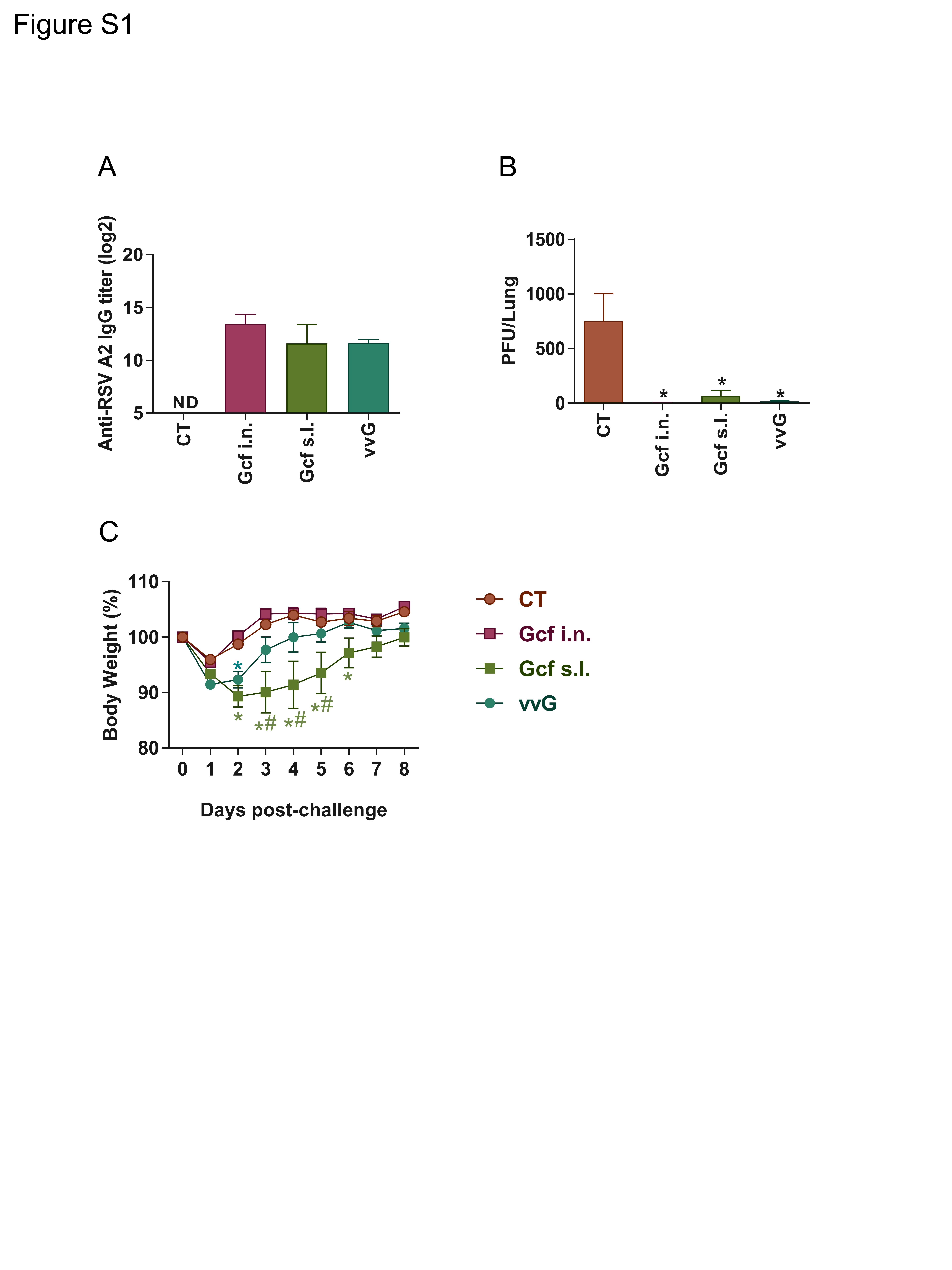

Supplement: Figure S1 — Anti-RSV immunity and enhanced disease in RSV challenged mice following sublingual Gcf immunization. (A) RSV-specific serum IgG titers measured at day 14 post-booster immunization by ELISA using 2 x 10^3^ RSV A2 per well as the coating antigen. Titers are indicated in log2. (B) Lung RSV titers at day 4 post-RSV A2 challenge determined by plaque assay. (C) Weight loss following RSV A2 challenge. Representative results of three independent experiments are shown. All data are expressed in mean ± S.E.M (n = 5). * indicates significant different with CT group (P < 0.05). # indicates significant difference with vvG group (P < 0.05). ND, Not detected. [file Image_1.TIF]

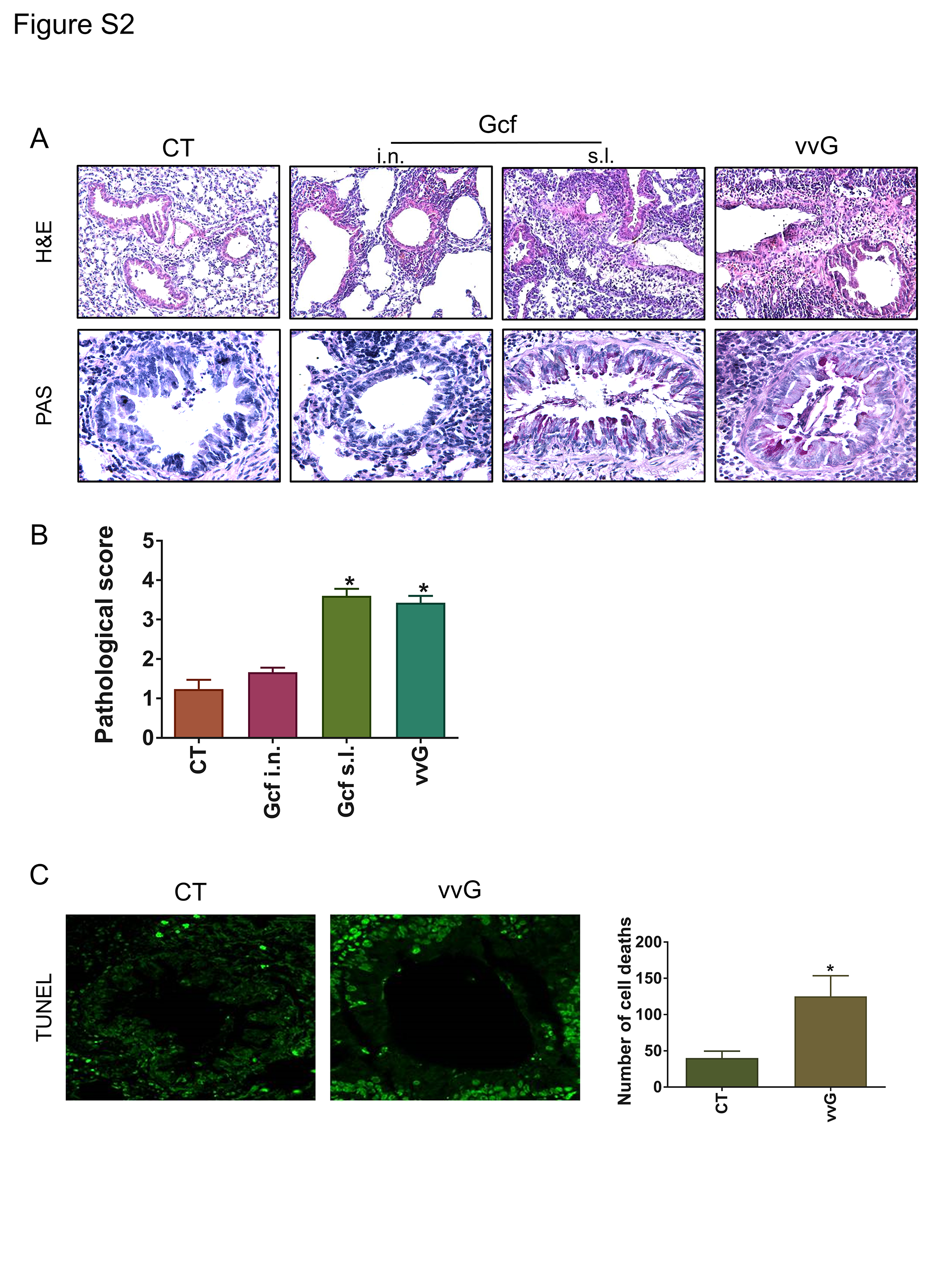

Supplement: Figure S2 — Lung histology of mice immunized with Gcf through different routes. All samples were collected at day 4 post-RSV challenge and prepared from formalin-fixed, paraffin-embedded lung tissues. H&E staining, TUNEL assay, and PAS staining were performed to determine inflammation, cell death, mucus secretion, respectively. (A) H&E staining (1st low, 20X magnification), and PAS staining (2nd low, 40X magnification) of lung samples harvested at day 4 post-challenge. (B) Lung pathological score assigned representing the inflammatory cell infiltration shown in H&E staining. (C) TUNEL+ cells (40X magnification) and quantification of cell death represented by TUNEL+ cells. Representative results of three independent experiments are shown. All data are expressed in mean ± S.E.M (n = 3). * indicates significant difference with CT group (P < 0.05). [file Image_2.TIF]

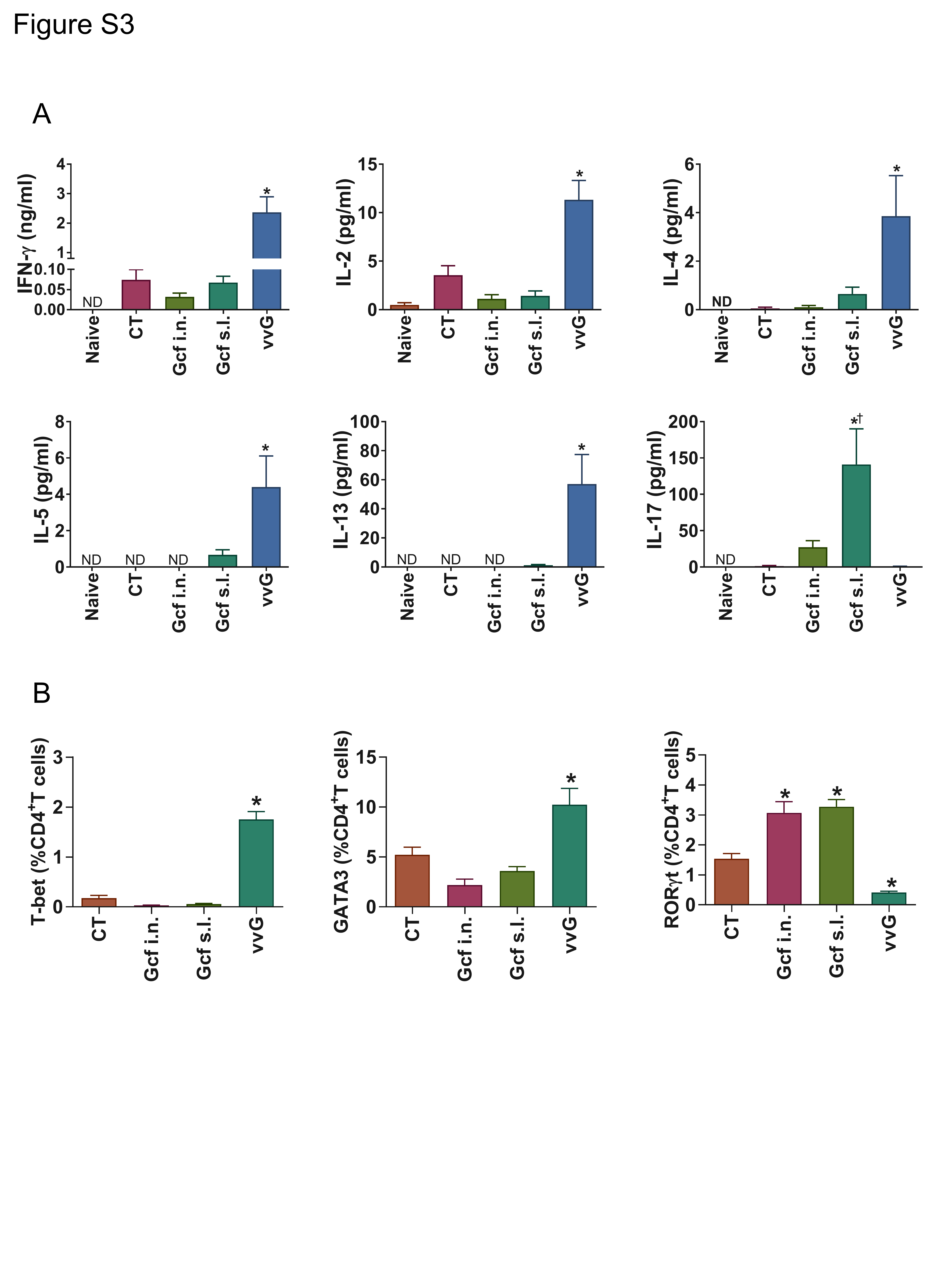

Supplement: Figure S3 — Cytokine profiles and transcription factor expression levels in the mice immunized with Gcf. (A) IFN-γ, IL-2, IL-4, IL-5, IL-13, and IL-17 levels in BAL fluid of mice immunized with Gcf at day 4 post-RSV A2 challenge determined by CBA. (B) Percentage of T-bet, GATA-3, or RORγt expressing splenic CD4+ T cells at day 7 post-booster immunization determined by intracellular staining. Representative results of three independent experiments are shown. All data are expressed in mean +/- S.E.M (n = 6). * indicates significant difference with CT group (P < 0.05). † indicates significant difference with Gcf i.n. group (P < 0.05). ND, Not detected. [file Image_3.TIF]
